# Smoking Cessation and Hospitalized Patients: A Missed Opportunity to Avoid Premature Deaths

**DOI:** 10.31486/toj.20.0095

**Published:** 2021

**Authors:** Roger J. Zoorob, Robert S. Levine, Charles H. Hennekens

**Affiliations:** ^1^ Department of Family and Community Medicine, Baylor College of Medicine, Houston, TX; ^2^ Affiliate Professor, Charles E. Schmidt College of Medicine, Florida Atlantic University, Boca Raton, FL; ^3^ First Sir Richard Doll Professor and Senior Academic Advisor to the Dean, Charles H. Schmidt College of Medicine, Florida Atlantic University, Boca Raton, FL

**Dedication**

We dedicate this manuscript to the memory of the late Edward D. Frohlich, MD, PhD, a great man and a good man who was a valued mentor, colleague, and friend. He was a member of the Ochsner family for more than 40 years, and his remarkable contributions helped propel Ochsner to become a premier research institution nationally and internationally.

## INTRODUCTION

Professor Sir Richard Doll, in collaboration with Professor Sir Austin Bradford Hill, were the first to quantitate the relationship of cigarette smoking with lung cancer.^1^ Later, Doll aptly stated, “Death in old age is inevitable, but death before old age is not.”^1^ The Richard Doll Building at the University of Oxford in Oxford, England, is inscribed with one of Doll's many impactful statements about cigarette smoking:

In previous centuries, 70 years used to be regarded as humanity's allotted span of life, and only about one in five lived to such an age. Nowadays, however, for non-smokers in Western countries, the situation is reversed: only about one in five will die before 70, and the non-smoker death rates are still decreasing, offering the promise, at least in developed countries, of a world where death before 70 is uncommon.^1^

Despite this evidence-based optimism, cigarette smoking and the emerging clinical and public health challenges of overweight and obesity and physical inactivity are the leading avoidable causes of premature death in most industrialized nations. These risk factors are also emerging in the rest of the world, so that smoking, overweight and obesity, and physical inactivity are the major contributors to the reason that cardiovascular disease (CVD) has increased from number 5 to number 1 as the cause of mortality worldwide.^2^

In the United States during the past several decades (since 1965), the prevalence of cigarette smoking has decreased markedly.^3^ Nonetheless, in 2018, approximately 34.2 million Americans aged 18 years and older, or 13.7% of the population, were current cigarette smokers.^3^ Thus, the impact of cigarette smoking on avoidable and premature mortality remains alarmingly high. In the United States alone, smoking causes more than 480,000 deaths each year.^3^ These sobering statistics reflect, in major ways, the approximate 2-fold increased risk of CVD among current smokers and the approximate 20-fold increased risk of lung cancer among long-term smokers. Specifically, these relative increases translate to absolute increases of more than 150,000 deaths per year from CVD and 130,000 deaths per year from lung cancer among persons ≥35 years of age.^4^ Quitting smoking before age 40 years reduces the risk of dying from cigarette-related disease by approximately 90%.^5^ Specifically, smoking cessation significantly reduces the risks of CVD—beginning within a matter of months—and the risk of CVD among those who quit smoking equals that of lifelong nonsmokers within a few years, even among older adults.^6^ In contrast, reductions in mortality risk from lung cancer only begin to appear several years after quitting, and even by 10 years, the risk has been reduced to only approximately midway between continuing smokers and lifelong nonsmokers.^7^

In the United States in 2016, approximately 30 million hospital admissions occurred among persons 18+ years of age, with an average length of stay of 4.6 days.^8^ A 2009 study in San Francisco reported the prevalence of cigarette smoking among hospitalized patients to be 40%^9^ in contrast to the prevalence in the general population of 13.7%.^3,10^ This prevalence implies that up to 12 million inpatients in US hospitals are smokers. In this paper, we discuss effective and safe counseling and drug therapies that have the potential to prevent numerous premature deaths in the United States caused by cigarettes in hospitalized smokers.

## SMOKING CESSATION COUNSELING

A systematic review of smoking cessation counseling studies examined 340 peer-reviewed publications of intensive smoking cessation programs for hospitalized patients and found that 326 (95.9%) did not meet quality standards.^11^ Among the remaining 14 studies, 8 showed no difference between the intervention and comparison groups.^12-19^ Three studies included self-reported and biochemically tested abstinence and reported positive results based on self-reports but not on biochemical testing.^20-22^ Two other studies reported positive results based on self-reports but did not include biochemical testing.^23,24^ However, a trial that randomized hospitalized adult smokers, identified by their desire to quit, to intensive vs standard counseling during hospitalization and after discharge resulted in higher rates of smoking cessation at 6 months in the intensive counseling group.^25^

For the intensive counseling group, postdischarge interventions included automated telephone calls and free medication. Prescription drug therapy was individualized for the intervention group, and patients received 5 automated outbound interactive voice response telephone calls at 2, 14, 30, 60, and 90 days after discharge. These telephone calls provided advice and support messages prompting smokers to stay quit, encouraging proper use and adherence to cessation medication, offering medication refills, and triaging patients who needed live support from counselors. An automated telephone script reinforced these messages and encouraged participants to request a callback from a counselor if they had low confidence in their ability to stay quit, had resumed smoking but still wanted to quit, needed a medication refill, or were noncompliant. The drug or drugs prescribed were documented in the medical record to alert the attending physician, and a fax was sent to the patient's primary care clinician as well. In contrast, standard care included postdischarge recommendations, advice to call a free telephone quit line, and a note in the medical record advising hospital physicians to prescribe smoking cessation medication at discharge.^25^

Short- and long-term results were based on biochemically confirmed abstinence at discharge and at 6-month follow-up. At 6 months, 26% of patients in the intensive counseling group had stopped smoking vs 15% of patients in the standard counseling group.^25^ This difference was highly significant but more important was its clinical and public health significance. Specifically, this relative reduction could translate into an absolute reduction of 1.32 million cigarette smokers among hospitalized patients in the United States.^3,8-10^

## DRUG THERAPIES

The US Food and Drug Administration (FDA) has approved 7 prescription and over-the-counter drug therapies for smoking cessation.^26^ Perhaps the most effective is the prescription medication varenicline that achieved permanent quit rates at 12 weeks of approximately 25% and was approved by the FDA in 2006. In 2009, however, varenicline received a black box warning based on reports to the FDA Adverse Event Reporting System (AERS) of neuropsychiatric symptoms including aggression, depression, and suicidal ideation. Data from the AERS are useful for formulating but not for testing hypotheses, and analysis showed that nearly half of the subjects had psychiatric histories, 42% were taking psychotropic drugs, and 42% had depression.^27^

The adverse public health impact of the black box warning was substantial, leading to a 76% decline in the number of prescriptions dispensed, from a peak of approximately 2 million in the last quarter of 2007 to approximately 531,000 in the first quarter of 2014.^27^ Gaballa et al estimated that because of the decrease in prescription rates, an estimated 17,000 annual US deaths from CVD attributable to smoking were avoidable between 2009 and 2016.^27^

In 2016, the FDA removed the black box warning, based, in large part, on the results of the Evaluating Adverse Events in a Global Smoking Cessation Study (EAGLES), a randomized trial of adequate size and 12 weeks’ duration.^28^ With respect to efficacy, varenicline was statistically significantly superior to bupropion and the nicotine patch, and bupropion and the patch were superior to placebo. Regarding side effects, varenicline produced no significant increases in serious neuropsychiatric symptoms in either the general population or patients with mental illness. The finding among patients with mental illness was especially important because the lifespan of patients with schizophrenia is reduced by approximately 20% compared to the general population.^29^ Although high premature death rates in patients with schizophrenia had been attributable to their 10-fold increase risk of suicide, patients with schizophrenia have a 40% or greater death rate from CVD that is attributable, in large part, to the approximate 74% rate of cigarette smoking among patients with schizophrenia, combined with overweight and obesity and physical inactivity.^29^

## BENEFIT OF MULTIFACTORIAL INTERVENTIONS

Providing multifactorial intensive counseling interventions during and after hospitalization and initiating and maintaining adherence to drug therapy are independently associated with permanent cessation rates.^30^ Among 9,193 smokers hospitalized for myocardial infarction and identified by the largest US registry, 97% received smoking cessation counseling during hospitalization, but only 7% filled their smoking cessation prescription within 90 days of discharge, and by 1 year, the percentage had only increased to 9.4%.^31^ In a Veterans Affairs hospital network, only 33.7% of patients with chronic obstructive pulmonary disease were prescribed a smoking cessation medication, and among these patients, 53.4% received nicotine patches alone.^32^ In another study involving 36,675 patients with coronary heart disease, only 22.7% (8,316) received any smoking cessation pharmaceutical during the hospitalization.^33^

Implementation of effective and safe antismoking campaigns has been suboptimal in communities as well as in hospitals. For example, among an estimated 53,107,842 active smokers with chronic obstructive pulmonary disease, the average prescription rate for smoking cessation efforts was 3.64%.^34^ Among smokers with peripheral artery disease, approximately 64% received no counseling or pharmacotherapy for smoking cessation.^35^

In theory, achieving 90% coverage with smoking cessation programs would save approximately 1,300,000 quality-adjusted life-years.^36^ This estimate is greater than the combined effects of screening for breast, colon, and cervical cancers; chlamydia; cholesterol; problem drinking; and vision.^36^ The health care system in the United States is characterized by numerous high-cost, low-value services such as baseline laboratory tests for low-risk patients having low-risk surgery ($227.8 million per year in unnecessary costs); stress cardiac or other cardiac imaging in low-risk asymptomatic patients ($93.2 million); annual electrocardiograms or other cardiac screening for low-risk asymptomatic patients ($41.0 million); and routine head computed tomography scans for emergency department visits for patients with dizziness ($24.8 million).^37^ In contrast, smoking cessation programs are cost-effective, in part, because the benefits to the economy far exceed the monetary value of the delivery of the effective multifactorial interventions.^36,38^

## CONCLUSION

The totality of evidence suggests that initiation of long-term counseling and adjunctive drug therapy during hospitalization and maintaining high adherence postdischarge can markedly improve permanent quit rates with minimal to no side effects. Programs should include long-term counseling and at least a 90-day prescription of a smoking cessation medication, preferably varenicline. Such efforts have the potential to reduce the number of avoidable premature deaths from cigarette smoking that remains alarmingly and unnecessarily high in the United States and worldwide.
